# Malignant Transformation and Metastatic Spread of Dumbbell-Shaped Meningeal Melanocytoma of the Cervical Spine: A Case Report and Literature Review

**DOI:** 10.3389/fsurg.2022.789256

**Published:** 2022-03-23

**Authors:** Shuang-lin Deng, Yu-bo Wang, Dan-hua Wang, Shuang Zhan, Yi Jing, Yi Guan

**Affiliations:** ^1^Department of Oncological Neurosurgery, First Hospital of Jilin University, Changchun, China; ^2^Department of Pathology, First Hospital of Jilin University, Changchun, China

**Keywords:** meningeal melanocytoma, melanoma, dumbbell-shaped tumor, malignancy, cervical spine

## Abstract

**Background:**

Meningeal melanocytoma is a rare disease that originates from leptomeningeal melanocytes in the central nervous system. Meningeal melanocytoma is generally considered benign, and has a good prognosis following complete surgical resection. Reports of the malignant transformation and spread of these tumors are scarce.

**Case Presentation:**

A 19 year old female presented with headache, progressive limb weakness, and dyspnea. Magnetic resonance imaging showed a dumbbell-shaped lesion at C1–C2 that was hyperintense on T1 weighted images and showed strong contrast enhancement. Total resection was achieved using a posterior midline approach. Post-operative pathology showed meningeal melanocytoma. The tumor recurred 9 months later with intracranial spread. Resection of the lesion revealed malignant transformation to meningeal melanoma.

**Conclusion:**

Meningeal melanocytoma harbors malignant potential even with total resection. Radiotherapy could be considered to prevent disease recurrence and progression.

## Background

Primary meningeal melanocytoma of the central nervous system (CNS) is rare, usually located at the ventrolateral medulla and cervical spinal cord (1), causing local compression. Compared to primary or secondary CNS melanoma, meningeal melanocytoma is generally considered benign, with potential for cure following complete surgical resection. Reports of malignant transformation and spread of meningeal melanocytoma of the CNS are scarce. To the authors' knowledge, there has been only one report of a case of spinal primary meningeal melanocytoma that underwent recurrence with malignant transformation after gross total resection (2). Here, we present the case of a primary meningeal melanocytoma recurring with malignant transformation and intracranial spread, and a review of the literature.

## Case Presentation

**Figure 1:**
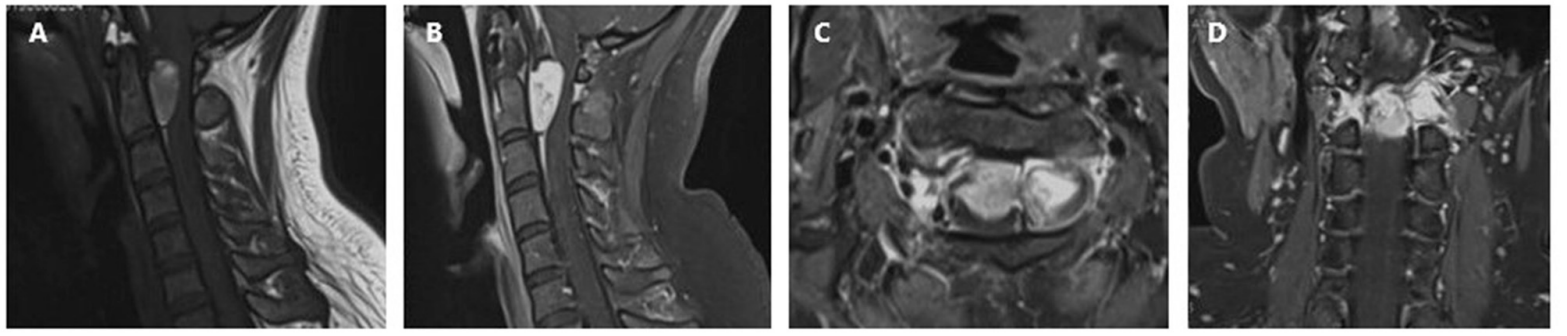
Preoperative cervical MRI prior to the first surgery. **(A)** Sagittal T1 weighted image showing a hyperintense lesion at C1–C2. The lesion is triangular shaped with a relatively clear margin, causing compression of the cervical spinal cord. **(B)** Sagittal contrast-enhanced T1 weighted image showing relatively homogenous enhancement. **(C)** Axial contrast-enhanced T1 weighted image showing the lesion extending from the subdural space, through the neuroforamen, forming a dumbbell-shaped contour. **(D)** Coronal contrast-enhanced T1 weighted image.

A 19 year old previously healthy female was admitted to our department with gradual onset headache, which lasted for 1 month, dyspnea, and progressive upper and lower limb weakness. The headache was prominent at frontal and occipital locations and worsened in the morning, the patient also experienced several episodes of “inability to breath” with increasing frequencies before admission. Neurological examination showed neck stiffness, tachypnea, and reduced muscle strength in the lower (3/5) and upper limbs (4/5) with normal muscle tone and sensory modality, pathologically exaggerated patellar and ankle reflexes were present. Laboratory tests were unrevealing except for mild hypoalbuminemia (37.2 g/L), and blood gas suggesting hyperventilation (PO_2_ 138.70 mmHg, PCO_2_ 34.00 mmHg). Cervical magnetic resonance imaging (MRI) revealed a dumbbell-shaped lesion with a relatively clear border (2.7 cm × 1.1 cm × 2.8 cm) compressing the spinal cord at C1–C2, and extending to the extra spinal space through the neural foramen. The lesion was hyperintense on T1 weighted images and had a mixed signal on T2 weighted images, with strong and relatively homogenous enhancement (Figure 1). Craniospinal MRI and whole-body positron emission tomography—computed tomography (PET-CT) excluded anomalies other than the lesion in the upper cervical spine. Skin examination excluded neurocutaneous melanosis.

### First Surgery and Pathology

**Figure 2:**
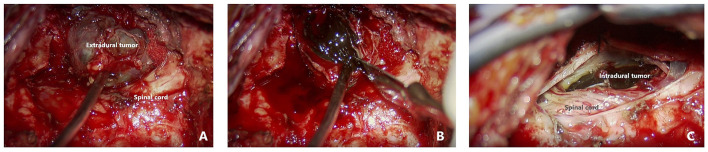
Intraoperative findings ***. **(A)** Intraoperative view of the extradural portion of the tumor. **(B)** Opening the capsule to reveal a blackish tumor. **(C)** Intradural portion of the blackish tumor anterior to the cervical cord.

**Figure 3:**
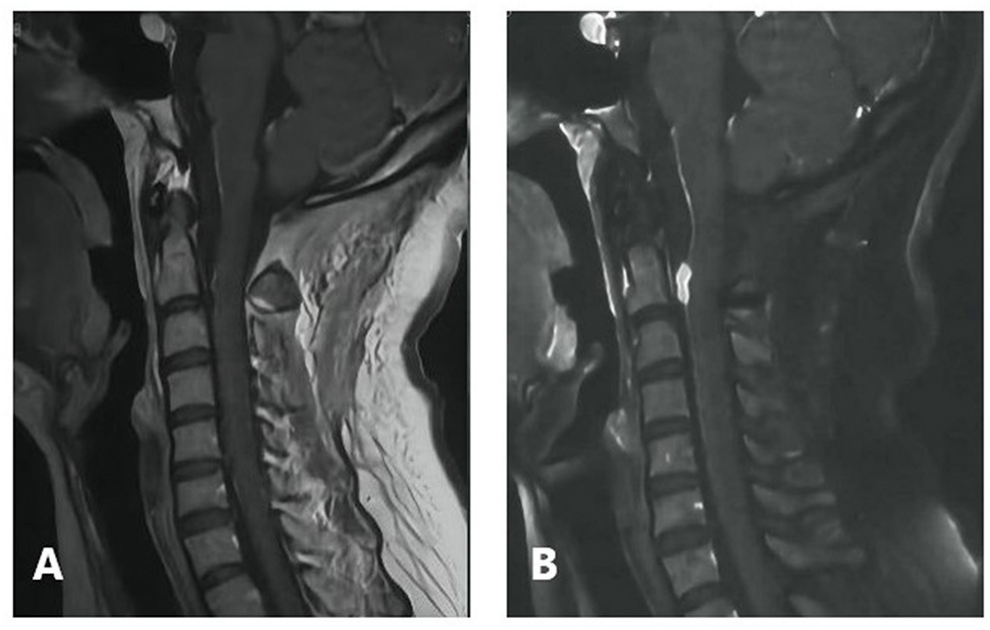
Post-operative follow-up. **(A)** No recurrence at 3 months **(B)** early local recurrence at 6 months.

A posterior midline approach was performed to expose a blackish mass with a rich blood supply located within the nerve sheath on the left side of the joint space between C1 and C2. The mass extended laterally to approach the vertebral artery and internally through the spinal foramen to enter the subdural space (Figure 2). Following incision of the nerve sheath, the mass was carefully isolated from peripheral tissue, with relatively clear margins. The mass was attached to the C1 nerve root, which was compressed; therefore, a portion of the nerve tissue was sacrificed. A longitudinal incision in the dura exposed the intra-dural portion of the tumor, which had a well-defined margin but displaced the spinal cord posteriorly. The dural attachment of the tumor was coagulated, and both the intra-dural and intraforaminal portion of the tumor were removed with en bloc resection. After surgery, the patient's symptoms were completely resolved. Post-operative MRI examination found no residual tumor, and no sign of recurrence at 3 months of follow-up (Figure 3).

**Figure 4:**
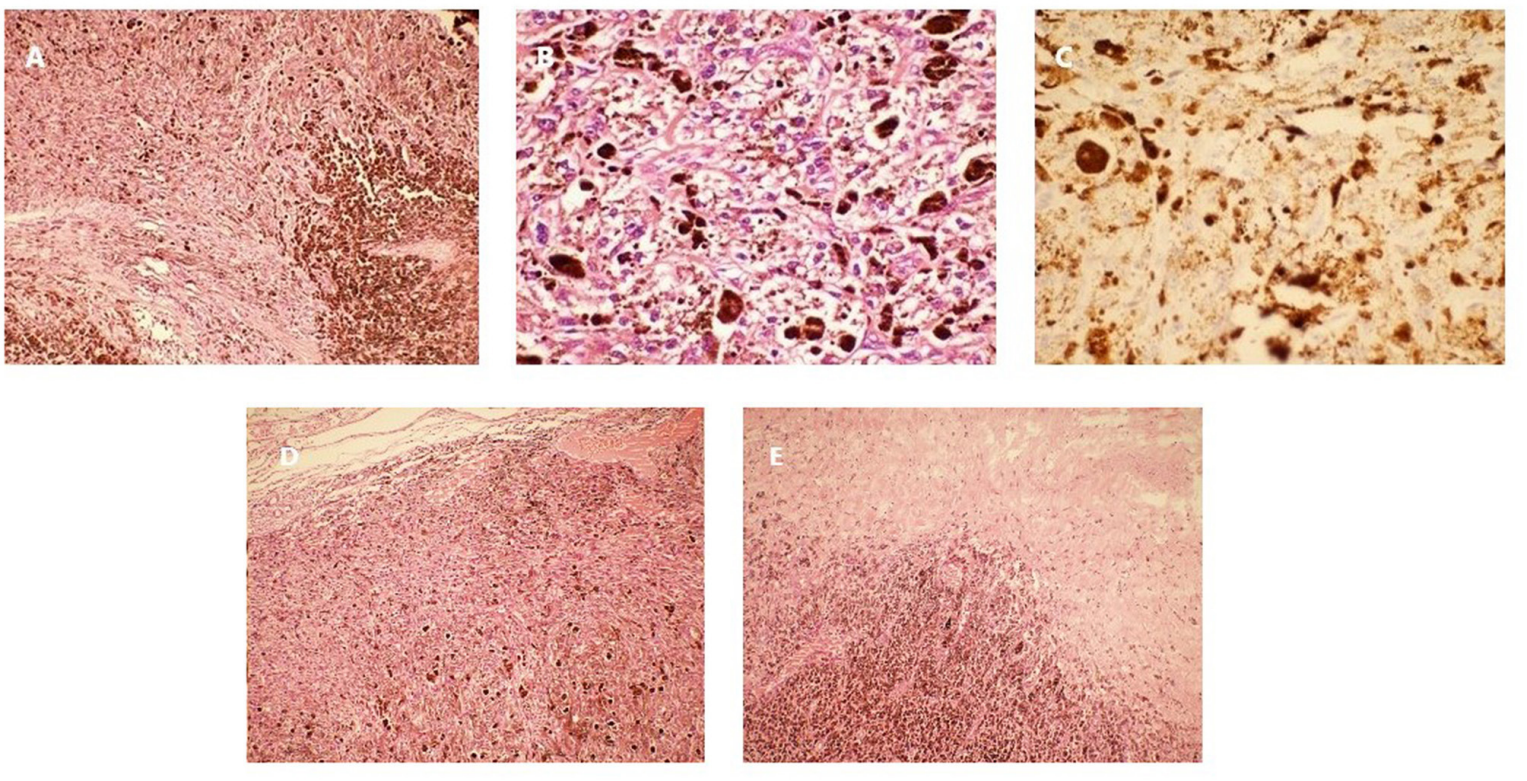
Pathology of the lesion resected during the first surgery. **(A)** Hematoxylin and eosin staining (x100). **(B)** Hematoxylin and eosin staining (x400). **(C)** Ki-67 immunostaining (x400). **(D)** Tumor pial junction (x100). **(E)** Tumor dural junction (x100).

Post-operative microscopic examination of the resected lesion revealed oval, short-spindle shaped cells with a heterogeneous intracellular distribution of melanin (Figure 4). High (x400) magnification showed oval cells with large nuclei, prominent nucleoli, and little mitotic activity (Figure 4). Immunostaining was cyclin D1, CD117, S-100, human melanoma black (HMB) 45, P53 and vimentin positive, and CD34, epithelial membrane antigen (EMA) and progesterone receptor (PR) negative. The MIB labeling index was 1–2% per slide (Figure 4), and no pial (Figure 4) or dural invasion (Figure 4) was seen. The definitive diagnosis was primary meningeal melanocytoma of the cervical spine.

### Second Surgery and Pathology

**Figure 5:**
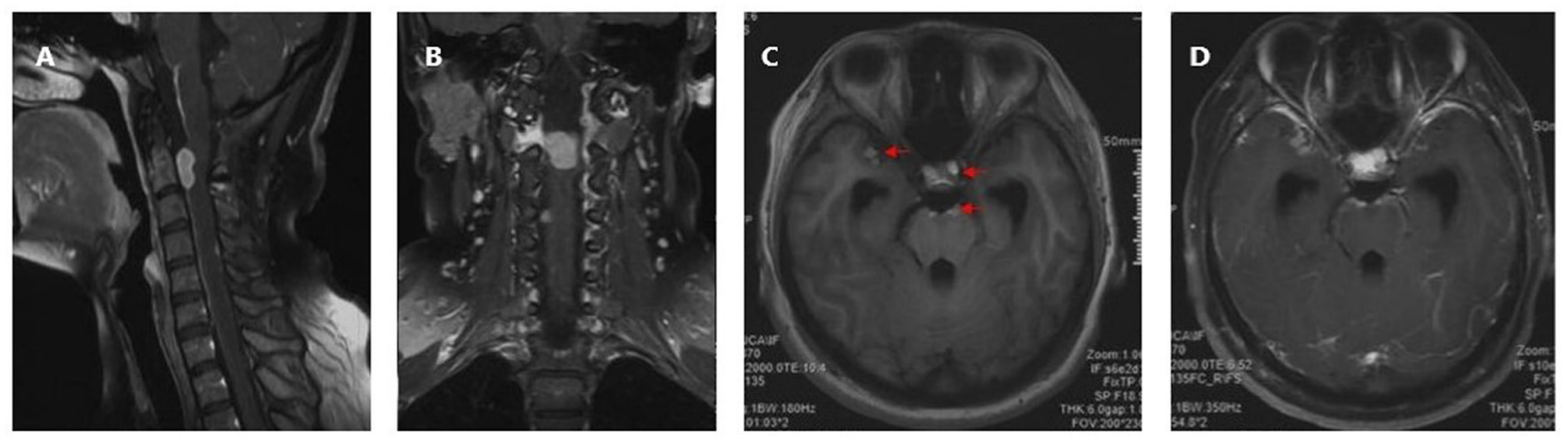
Preoperative head and cervical MRI prior to the second operation. **(A)** Sagittal contrast-enhanced T1 weighted image. **(B)** Coronal contrast-enhanced T1 weighted image. **(C)** Axial T1 weighted image showing intracranial spread (Red arrow). **(D)** Axial contrast-enhanced T1 weighted image showing metastasis (enhanced).

Six months after the initial surgery there was early local recurrence of the tumor at the initial site (Figure 3). A watch and wait strategy was adopted based on the initial benign pathology and because the tumor was located in the upper cervical spine. Radiotherapy was postponed with close observation. However, 3 months later the patient was readmitted for severe headache and vomiting. Head and cervical MRI revealed a mass (~2.0 cm in diameter) near the initial surgical site that was hyperintense on T1 weighted images and showed strong enhancement. There was metastasis at C5, intracranial spread at the left temporal pole, supra-sellar cistern, pre-pontine cistern, sellar and ventral pontine region, with concurrent obstructive hydrocephalus. The medulla appeared hypointense on T1 weighted images and hyperintense on T2 weighted images (Figure 5). Thoracic and abdominal CT scan did not reveal extra-cranial and extra-spinal metastasis. As the patient developed severe headache mostly related to obstructed circulation of the cerebrospinal fluid, a palliative excision of the lesion near the initial surgical site was performed. The tumor had a similar appearance but a more extensive blood supply than the initial lesion. The tumor displaced the spinal cord and was firmly attached at its ventral surface. The patient underwent subtotal resection and experienced partial symptom relief.

**Figure 6:**
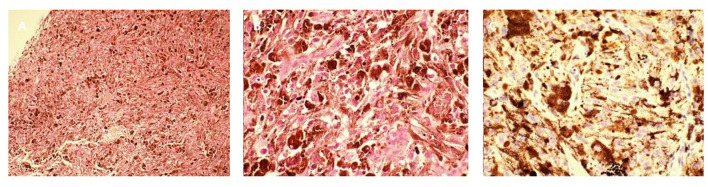
Pathology of the lesion resected during the second surgery. **(A)** Hematoxylin and eosin staining (x100). **(B)** Hematoxylin and eosin staining showing multiple anaplastic cells and mitotic figures (x400). **(C)** Ki-67 immunostaining (x400).

Microscopic examination of the resected lesion confirmed melanoma (Figure 6). High (x400) magnification showed extensive anaplasia and mitotic activity (Figure 6). The MIB labeling index was 5–10% (Figure 6). Genetic analysis of the pathology showed frame shift mutation in PRKAR1A (c.351del), missense mutation in RPS6KB2 (c.1444C>T), and SERPINB4 (c.449G>T), but no mutation in BRAF and KIT or other mutations as available therapeutic targets. The patient harbored germ-line heterozygote mutation in WT1 (c.199C>T) which was likely pathogenic. The tumor mutation burden was 1.41/Mb, and Microsatellite Instability was stable.

The analysis suggested limited response to immuno-chemotherapy, and no available targeted therapy, yet considering cancer spread in the central nervous system could be quickly lethal, experimental treatment with dynamic adjustment to response was agreed with the patient and her family. The therapy started with 1 cycle of Pembrolizumab+Bevacizumab+Temozolomide. Radiation therapy was scheduled but the patient developed surgical site CSF leak, hence was postponed. Upon re-evaluation with cranial-spinal MRI, intracranial metastasis showed enlargement hence chemotherapy regimen was modified to replace Temozolomide with Ipilimumab considering some previously reported limited data, suggesting a potential benefit of CTLA4 antibody therapy for melanoma carrying SERPINB4 mutation. The patient achieved stable disease after 2 cycles of Pembrolizumab+Bevacizumab+Ipilimumab, yet developed progressive disease again after the third cycle. Experimental therapy replacing bevacizumab with Anlotinib and Paclitaxel for two cycles did not achieve a response. The patient underwent craniospinal irradiation (planned dose: 30 Gy/15f, actual dose tolerated: 20 Gy/10f), then followed with 2 cycles of intravenous Pembrolizumab+Bevacizumab+Ipilimumab and intrathecal Pembrolizumab, and 13 cycles of intravenous Pembrolizumab+Bevacizumab and intrathecal Pembrolizumab, during which the disease remained stable but progressed upon re-evaluation after the 14th cycle, and the patient demised 2 months later.

## Discussion and Conclusion

In 1972, Limas and Tio described the case of a 71-year-old male who presented with a black, smooth-surfaced mass that was not attached to the dura at the craniocervical junction. The mass was discovered on autopsy after a long and progressive clinical course (3). Histology and ultrastructure excluded a diagnosis of “pigmented' or “melanotic” meningioma, and the term “meningeal melanocytoma” was proposed. Similar to primary melanoma of the CNS, primary meningeal melanocytomas of the CNS originate from melanocytes of the leptomeninges and are usually located at the ventrolateral medulla and the cervical spinal cord (1). They may also arise at the supra- and infratentorium regions, cerebellopontine angle, and sellar region (4–8), and other regions of the spine at both extramedullary and intramedullary locations. Generally, primary meningeal melanocytoma of the CNS is rare, with <200 cases reported up to 2020, and an estimated incidence of one per 10 million individuals (9).

Extramedullary spinal meningeal melanocytoma often presents as a mass that causes signs and symptoms of spinal cord compression. For dumbbell-shaped tumors of the upper cervical spine that involve spinal roots, clinical manifestations may also include myelopathy and myeloradiculopathy, most of which had a course of disease within 1 year (Table 1). MRI can be used to determine the outline of the mass and the extent of spinal cord compression. In contrast to other tumors of the CNS, but similar to melanoma of the CNS, meningeal melanocytoma of the CNS often appears hyperintense on T1 weighted images and hypointense on T2 weighted images (17). As melanocytoma has a relatively homogenous histology, the tumor usually shows uniform contrast enhancement (18); although in some cases, the enhancement may be heterogeneous. In the present case, cervical MRI revealed a dumbbell-shaped extramedullary meningeal melanocytoma at the upper cervical spine. Based on lesion signal characteristics, the preoperative differential diagnosis included meningioma, and considering the contour of the tumor, a rare form of schwannoma was not excluded.

**Table 1:** Previously published reports of dumbbell-shaped spinal meningeal melanocytoma.

**Author and Year**	**Age/Gender**	**Location**	**Clinical manifestation**	**Course of disease**	**Pathology**	**Treatment**	**Nerve root**
Tatagiba et al. (10)	48/M	C7–T1, intervertebral foramen enlargement	Radicular pain	12 months	Meningeal melanocytoma	Complete resection	Preserved
Sankhla et al. (11)	19/M	C4, intervertebral foramen enlargement	Progressive weakness in the upper extremity, c5 dermatome paraesthesiae	4 months	Meningeal melanocytoma	Partial resection	Preserved
Goyal et al. (12)	33/F	L3–L4	Back Pain radiating left lower limb, progressively increasing weakness	3 years	Meningeal melanocytoma	Partial resection	L3 nerve root excised
El-Khashab et al. (13)	17/F	C5–C7 intervertebral foramen enlargement	Progressive extremity weakness and sensory disturbance	3 weeks	Intermediate grade meningeal melanocytoma	Partial resection	–
Bhargava et al. (14)	55/M	C1–3 foramen enlargement	Numbness and progressive weakness	1 year	Meningeal melanocytoma	Subtotal resection	Preserved
Foit et al. (15)	43/M	C2–3/T1–2	Neck and shoulder pain, cephalalgia	4 months	Meningeal melanocytoma	Partial resection	Preserved
Miura et al. (16)	40/M	C2–C3	Numbness and hemipares	2 months	Meningeal melanocytoma	Complete resection	R3 nerve root excised

The most common treatment for spinal meningeal melanocytoma is total surgical resection. In one review of 89 meningeal melanocytomas of the CNS, the 5 year local control rate was 80% after complete resection, but only 18% after incomplete resection (19). Most spinal meningeal melanocytomas are extramedullary and located in the sub-meningeal space; therefore, en-bloc resection can be achieved. In the present case, the dumbbell-shaped morphology of the tumor was a particular challenge. Dumbbell-shaped tumors are a surgical challenge as extension in a lateral direction through the neuroforamen provides limited exposure and close contact with nerve roots. Only 7 cases of dumbbell-shaped meningeal melanocytomas have been previously reported in detail (Table 1) (10–16). While gross total resection can be achieved for most spinal meningeal melanocytomas with normal morphology (20, 21), there have been only two previous reports of dumbbell-shaped spinal meningeal melanocytomas achieving a gross total resection. The Eden classification designates 4 types of dumbbell-shaped tumors and has been used to define surgical approaches, including anterolateral, posterior, or combined (22). The present case was classified as a Type 2 tumor, occupying the intradural, extradural and paravertebral spaces. Although the intraspinal and extraspinal components were comparable in size, a posterior approach was chosen as it was predicted to adequately expose the tumor and was familiar to our neurosurgeons. With slight lateral expansion, the extraspinal component could be isolated from adjacent tissue. With a clear margin between the tumor and the surface of the spinal cord, sacrifice of the heavily encroached nerve root enabled en bloc resection of the intraspinal component from the subdural space.

The spectrum of melanocytic neoplasms of the CNS ranges from meningeal melanocytoma, which are usually benign, to meningeal melanoma, which are malignant. According to the 2016 World Health Organization (WHO) classification of CNS tumors, melanocytomas have a Ki-67 index <1–2% and primary melanomas have a Ki-67 index of ~8%. In 1999, Brat et al. termed the transitional state between melanocytoma and melanoma, intermediate grade meningeal melanocytoma (1). According to Brat et al., classical features of a primary melanoma include cytological atypia, a high mitotic rate, or anaplasia. These features are lacking in intermediate grade meningeal melanocytoma. On immunohistochemistry, intermediate grade meningeal melanocytoma is usually HMB-45, S-100 and vimentin positive, which is similar to benign meningeal melanocytoma, but the former are believed to be more biologically aggressive. Brat et al. (1) estimated the MIB labeling index of intermediate grade meningeal melanocytoma as 1–4%, overlapping with the current criteria for CNS melanocytoma based on the 2016 WHO classification. Other studies have reported an MIB labeling index of 10% for “intermediate grade meningeal melanocytoma” (13, 23) and >5% for “meningeal melanocytoma with increased proliferation” (24). Due to the scarcity of available pathological data from these cases at a transitional state, an established cut-off value and diagnostic criteria are lacking. For primary CNS melanocytic tumors, the detailed biological pattern for the transformation from melanocytoma to intermediate grade lesion and eventually melanoma remains unknown. A literature review found 6 cases describing the malignant transformation of meningeal melanocytoma with detailed documentation of changes in the Ki-67 index (Table 2) (25–30). Except for one case, where malignant transformation was identified on the primary pathology examination, the duration from the initial confirmation of melanocytoma to the eventual transformation to melanoma ranged from 7 months to 5 years. In 4 of the 5 cases, this duration was longer than 2 years. Similar to a report by Roser et al. (26), the present case had a short disease course, with the Ki-67 index elevated from 1–2 to 10% in 9 months. Currently, it is unknown whether cases with a fast malignant transformation have a different biology compared to those with a longer course of disease.

**Table 2:** Previously published reports of meningeal melanocytoma with malignant transformation.

**Author and Year**	**Age/Gender**	**Location**	**Clinical manifestation**	**Primary Pathology/Ki-67**	**Treatment**	**Secondary pathology/Ki-67**	**Duration between last confirmed benign pathology to confirmed malignant transformation**
Uozumi et al. (25)	49/M	Left frontal lobe	Headache	Meningeal melanocytoma 0–1%	Total resection	Melanoma/5–10%	5 years
Roser et al. (26)	37/F	Petroclival area,	Progressive brain stem syndrome	Meningeal melanocytoma 5%	Subtotal resection	Melanoma/25%	7 months
Perrini et al. (27)	79/F	T10–T11	Progressive lower limb paresis	Intermediate grade meningeal melanocytoma 1–4%	Subtotal resection	Melanoma/15%	2 years
Wang et al. (28)	32/M	Right temporal lobe	Headache	Meningeal melanocytoma 1–2%	Total resection	Melanoma/20%	3 years
Gempt et al. (29)	71/F	Right frontal lobe	Speech and motor dysfunction	Meningeal melanocytoma 2%/ melanoma 12%	Partial resection	–	–
Küsters-Vandevelde et al. (30)	43/F	Right parietal lobe	Headache	Intermediate grade meningeal melanocytoma 5%	Total resection	Melanoma 10%	32 months

The role of irradiation as an adjuvant therapy following complete resection of spinal meningeal melanocytoma remains controversial. Rades et al. reported a 5 year local control rate of 100% with complete resection plus radiation, compared with 80% with complete resection alone (19). Yang et al. (20) reported a 100% control rate at a median follow-up of 58.1 months with complete resection or two stage resection without radiotherapy (20). Hence indiscriminative adoption of radiotherapy for cases achieving gross total resection is difficult to justify. Recurrence with malignant transformation and distant spread following an initial complete resection, as in the present case, is an extremely rare condition. Two similar reports have been published. One case involved a meningeal melanocytoma of the lumbar spine recurring with subcutaneous seeding. The initial Ki-67 index was not reported but the pathological description conformed to that of a classic benign lesion (2). The other case involved an intracranial lesion diagnosed as a benign meningeal melanocytoma that showed local recurrence and malignant transformation to meningeal melanoma. The initial Ki-67 index was 1–2%, but rose to 20% upon recurrence (28). Taken together, these cases indicate that total resection of even a benign lesion may be associated with a risk of leaving microscopic tumor remnants that retain the potential for malignancy. These remnants may result in local re growth or spread via the cerebrospinal fluid circulation to distant locations. Currently, it is unknown which subgroup of patients with gross total resection carries a higher risk for recurrence and malignant transformation and will benefit from postoperative radiotherapy. Unfortunately for our case, based on the initial diagnosis and the achievement of total resection, such a quick recurrence with malignant transformation was not anticipated, hence a detailed genetic analysis was not carried out until the first recurrence with malignant transformation for therapeutic purpose. The patient and her family refused retrospective genetic analysis of the initial pathology, hence this limited the possibility to further answer the cause for quick malignant transformation on the molecular level. The analysis of the pathology at recurrence was mostly unproductive in identifying therapeutic targets besides SERPINB4 mutation. Somatic SERPINB3 and SERPINB4 mutations are associated with survival following anti-CTLA4 immunotherapy in melanoma patients in retrospective analysis (31), and in prospective study, 5 of the 6 patients carrying SERPINB3/4 mutation responded to PD-1 blockers (32). The patient in our case carried only SERPINB4 mutation, and achieved stable disease of short duration after addition of anti-CTLA 4 treatment. However, the longest duration of intracranial metastasis was more likely to be a response to intrathecal PD-1 blocker.

Genetic analysis also identified germ-line mutation in WT1 (c.199C>T). WT1 participate in prenatal development of kidneys and gonads with its action in cell differentiation and apoptosis. WT1 is reported to be expressed in more than 80% of malignant melanoma cells and is associated with shorter overall survival (33). Mutation of WT1 is notoriously known to be associated with a series of oncological diseases including Wilms tumor. Its association with melanoma has also been documented, mostly somatic mutation. The patient in our case carried WT1 heterozygote germline non-sense mutation (c.199C>T), this association with melanoma has not been reported, and whether it contributed to the early recurrence with malignant transformation despite total resection, remains more data accumulation and further study.

In conclusion, even though most meningeal melanocytomas achieve a favorable outcome following a complete resection, the risk of recurrence, malignant transformation and distant metastasis still exist. Considering the poor prognosis, radiotherapy following a complete resection might be an option for selected cases. Further study is needed to identify patients with higher risk of recurrence and malignant transformation to ensure individualized management.

## Data Availability Statement

The raw data supporting the conclusions of this article will be made available by the authors, without undue reservation.

## Ethics Statement

Written informed consent was obtained from the participant for the publication of this case report (including all data and images).

## Author Contributions

S-lD designed most of the investigation, data analysis, and wrote the manuscript. Y-bW provided surgical assistance and participated in data analysis. D-hW provided pathological assistance. SZ provided neuroimaging assistance. YJ was responsible for the patient management and follow-up. YG performed the surgery and collected the clinical data. All authors have read and approved the manuscript.

## Funding

This work was supported by Scientific and Technological Planning Poject of Ji Lin Province—Jilin provincial Science Foundation—Project No. 20200201512JC.

## Conflict of Interest

The authors declare that the research was conducted in the absence of any commercial or financial relationships that could be construed as a potential conflict of interest.

## Publisher's Note

All claims expressed in this article are solely those of the authors and do not necessarily represent those of their affiliated organizations, or those of the publisher, the editors and the reviewers. Any product that may be evaluated in this article, or claim that may be made by its manufacturer, is not guaranteed or endorsed by the publisher.

## Abbreviations

CNS: central nervous system
EMA: epithelial membrane antigen
HMB: human melanoma black
MT: malignant transformation
MRI: magnetic resonance imaging
PET-CT: positron emission tomography-computed tomography.
